# Long Noncoding RNA MALAT1 Acts as a Competing Endogenous RNA to Regulate TGF-*β*2 Induced Epithelial-Mesenchymal Transition of Lens Epithelial Cells by a MicroRNA-26a-Dependent Mechanism

**DOI:** 10.1155/2019/1569638

**Published:** 2019-04-22

**Authors:** Ning Dong

**Affiliations:** Department of Ophthalmology, Beijing Shijitan Hospital, Capital Medical University, Beijing, China

## Abstract

The aim of the present study was to characterize whether the long noncoding RNA metastasis-associated lung adenocarcinoma transcript 1 (MALAT1)/miR-26a/Smad4 axis is involved in epithelial–mesenchymal transition (EMT) of lens epithelial cells (LECs). Primary human LECs were separated and cultured. Microarray analysis showed that a total of 568 lncRNAs are differentially expressed in primary HLECs in the presence of TGF-*β*2 and MALAT1 is mostly significantly dysregulated lncRNAs, which is increased by nearly 17-fold. In addition, upregulation of MALAT1 and downregulation of miR-26a were detected in human posterior capsule opacification (PCO) attached LECs and the LECs obtained from patients with anterior polar cataracts by quantitative RT-PCR (qRT-PCR). Next, our results showed that TGF-*β*2 induces overexpression of EMT markers in primary HLECs via a MALAT1-dependent mechanism. The mechanism is that MALAT1 negatively regulates miR-26a and miR-26a directly targets Smad4 by luciferase reporter assays and RNA-binding protein immunoprecipitation assay. In summary, TGF-*β*2 induces MALAT1 overexpression, which in turn MALAT1 acts as a ceRNA targeting Smad4 by binding miR-26a and promotes the progression of EMT of LECs.

## 1. Introduction

Posterior capsule opacification (PCO) is commonest postoperative complication of cataract surgery, which causes impairment of vision [1, 2]. After cataract surgery, the postoperative residual lens epithelial cells (LECs) undergo the pathological progression, including proliferation, migration, and epithelial–mesenchymal transition (EMT), which is an important cause of PCO formation [1–5]. Cumulative evidence reveals that EMT of LECs plays a central role in the pathogenesis of PCO. During EMT, LECs undergo loss of cells adhesion and apical–basal polarity, which results LECs transdifferentiate into mesenchyme-like cells [1–5].

It is well known that only ~2% of RNA is a blueprint for proteins, and the remaining ~98% serves as noncoding RNAs (ncRNAs) [6]. Long noncoding RNAs (lncRNAs) represents a novel class of RNAs which are longer than 200 nucleotides in length without functional protein-coding ability, which have attracted much attention recently [7]. Recent studies have suggested that the lncRNA metastasis-associated lung adenocarcinoma transcript 1 (MALAT1), located on chromosome 11q13, which is involved in the process of EMT in the cancer [8, 9]. Moreover, the emerging evidence has revealed a potential contribution of particular lncRNAs to the development of PCO [10, 11]. However, to the best of our knowledge, whether MALAT1 can regulate EMT of LECs and contribute to the pathogenesis of PCO has not been fully elucidated.

MicroRNAs (miRNAs) can regulate the posttranscriptional expression of protein-coding mRNAs [12, 13]. Mechanistically, miRNAs can block translation through binding to the 3' untranslated regions (UTRs) of target mRNAs [12, 13]. Our previous studies have demonstrated that miR-26b and miR-181a inhibit the proliferation, migration, and EMT of lens epithelial cells [3, 4]. In addition, increasing evidence indicates that miR-26a and miR-26b, which are members of the miR-26 family, have key roles in EMT of LECs [14]. Recently, a novel regulatory mechanism, competing endogenous RNAs (ceRNAs), in which RNAs can engage in cross-talk via competing for shared miRNAs, has been proposed [15, 16]. Our analysis, using the online software program starBase v2.0 (http://starbase.sysu.edu.cn/starbase2/index.php), predicted that microRNA-26a formed complementary base pairing with MALAT1 [17].

Therefore, the aim of the present study was to explore whether the lncRNA MALAT1/miR-26a axis is involved in EMT of LECs.

## 2. Materials and Methods

### 2.1. Patient Lens Epithelial Cell Collection and Culture

Patient LECs collection and culture have been described in detail previously [3–5]. Briefly, fresh lens capsules with adherent LECs were obtained from the Department of Ophthalmology, Beijing Shijitan Hospital, Capital Medical University (Beijing, China), during cataract surgery from 66 patients (66 eyes) with the clinical diagnosis of nuclear cataracts (33 patients; 33 eyes; 20 males and 13 females) or anterior polar cataracts (33 patients; 33 eyes; 18 males and 15 females) from June 2015 to December 2016. The ages of the patients ranged from 61 to 76 years. The inclusion criteria for both groups were free from other ocular diseases except cataract. The study was approved by the Ethics Committee of Beijing Shijitan Hospital and was performed in accordance with the Declaration of Helsinki. Each subject received a detailed information leaflet and provided informed written consent before participation. Fresh PCO tissues (16 organ donors; 16 eyes) and normal-attached LEC samples (15 organ donors; 15 eyes) were provided by the Eye Bank of Beijing, China (Beijing, China).

Primary human lens epithelial cells (HLECs) were cultured according to our previous procedure [4]. Primary HLECs were used to determine the role of lncRNA MALAT1/miR-26a axis in EMT of LECs.

### 2.2. SRA01/04 Cell Culture

Human lens epithelial cell line SRA01/04 was obtained from the Cancer Institute and Hospital, Chinese Academy of Medical Sciences (Beijing, China). The method for SRA01/04 cell culture was cultured according to our previous procedure [3–5].

SRA01/04 cells were only used for RNA immunoprecipitation (RIP) and the luciferase study.

### 2.3. Microarray Analysis

For Microarray analysis, the primary HLECs were treated with TGF-*β*2 (5 ng/ml) in 6-well plates for 48 h. Microarray analysis was performed by human lncRNA Array v2.0 (Arraystar, Rockville, MD, USA), which target differential expression of lncRNAs on the primary HLECs treated with TGF-*β*2 (experiment) and it treated without TGF-*β*2 (control).

### 2.4. Transfection

Small interfering RNAs (siRNAs) targeting MALAT1, including siMALAT1-1and siMALAT1-2, were obtained from GenePharma (Shanghai, China). Primary HLECs were transfected with 100 nM MALAT1 siRNAs or negative control siRNA [si-control] individually for 24 h. The siRNA sequences are shown in Table 1.

In addition, miR-26a mimics, anti-miR-26a, miR-26a mimics negative control, and anti-miR-26a negative control were obtained from GenePharma. The primary HLECs were transiently transfected with 100 nM miR-26a mimics or anti-miR-26a or negative control for 6 h using GenePORTER transfection reagent (GTS, Inc., San Diego, CA, USA).

The MALAT1 sequence was subcloned into the HindIII and EcoRI sites of pcDNA3.1 (Invitrogen, Carlsbad, CA, USA) vector, named pcDNA3.1-MALAT1. The MALAT1 sequence binding miRNA-26a response elements were mutated in which 5'-CUUGUUAUUUUUUACUUGA-3' changed to 5'-ACCACCCCCCCCCCACCAC-3'. The pcDNA3.1-MALAT1 with mutations was named pcDNA3.1-MALAT1-mut (miRNA-26a). The primary HLECs were transfected with pcDNA3.1-MALAT1 and pcDNA3.1-MALAT1-mut in order to achieve the ectopic expression of MALAT1. The primary HLECs were transfected with an empty pCDNA3.1 vector used as a control.

### 2.5. Quantitative Reverse Transcription PCR (qRT-PCR)

qRT-PCR was performed according to our previous procedure [3–5, 12, 13]. qRT-PCR primers are shown in Table 2.

### 2.6. Western Blot Analysis

The primary antibodies, including anti-E-cadherin (Abcam, Cambridge, MA, USA), anti-fibronectin (Abcam), anti-Smad4 (Abcam), and anti-actin (Abcam) were used for western blot analysis. The western blot analysis was performed as described our previously [3–5].

### 2.7. Luciferase Assay

Luciferase assay was performed according to our previous procedure [3–5]. The 3'-UTR of Smad4 mRNA and lncRNA MALAT1 containing the predicted miR-26a binding sites or corresponding mutant sites were amplified by PCR. Reporter activities were assessed 24 h after transfection using the dual-luciferase reporter assay system (Promega, Madison, WI, USA).

### 2.8. RNA-Binding Protein Immunoprecipitation Assay

RNA immunoprecipitation (RIP) was performed using an EZ-Magna RIP RNA-binding protein immunoprecipitation kit (Millipore, Billerica, MA, USA) according to our previous procedure [18].

### 2.9. Statistical Analysis

All data are presented as the mean ± SE. The SPSS for Windows Version 17.0 (SPSS, Inc., Chicago, IL, USA) software was used for statistical testing [18]. Student's t test was used to determine differences between two independent groups [18]. One-way analysis of variance (ANOVA) and post hoc test of Tukey's multiple comparisons were used to determine differences among multiple groups [18].* P* < 0.05 was considered statistically significant.

## 3. Results

### 3.1. TGF-*β*2 Induces the Different Expression of LncRNAs in Primary HLECs

**Figure 1:**
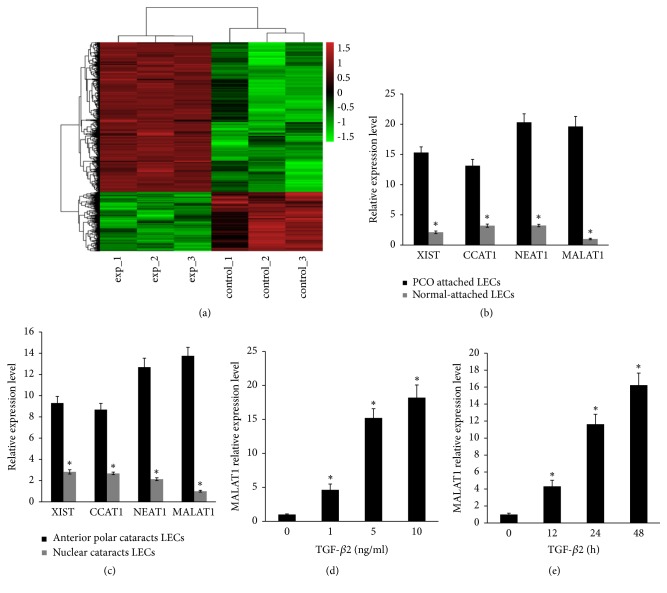
TGF-*β*2 induces different expression of lncRNAs in primary HLECs. (a) Heat map showed the mostly significant differential expression of lncRNAs in Primary HLECs by human lncRNA Array v2.0. The primary HLECs were treated with TGF-*β*2 (5 ng/ml) for 48 h which were experiment groups. The primary HLECs were treated without TGF-*β*2 which were control groups. (b) The expression of XIST, CCAT1, NEAT1, and MALAT1 was tested in human PCO attached LECs (n=16) and normal attached LECs (n=15) by qRT-PCR. *∗P *< 0.05 compared with PCO attached LECs. (c) The expression of XIST, CCAT1, NEAT1, and MALAT1 was detected in LECs obtained from patients with anterior polar cataracts (n=33) and patients with nuclear cataracts (n=33) using qRT-PCR. *∗P *< 0.05 compared with anterior polar cataracts. (d) qRT-PCR showed that the indicated concentration of TGF-*β*2 induces over-expression of MALAT1 in primary HLECs in a dose-dependent manner. *∗P *< 0.05 compared with group without TGF-*β*2. (e) qRT-PCR showed that the indicated time of 5 ng/ml TGF-*β*2 induces overexpression of MALAT1 in primary HLECs in a time-dependent manner. *∗P *< 0.05 compared with group without TGF-*β*2. (b, c, d, and e) The data are presented as the mean± SE of six independent experiments.

TGF-*β* is considered to be a crucial inducer of EMT-related changes in PCO and anterior subcapsular cataracts [19]. TGF-*β*2, which is one of TGF-*β* superfamily, has been proposed as the major isoform within the aqueous humor [20, 21]. To identify the involvement of lncRNAs in PCO development, we analyzed the different expression of lncRNAs in primary HLECs in the presence or absence of TGF-*β*2 using human lncRNA Array v2.0. The heat map was used to show that lncRNAs were differentially expressed between primary HLECs treated with 5 ng/ml TGF-*β*2 (experiment) and it treated without TGF-*β*2 (control) (Figure 1(a)). A total of 568 lncRNAs exhibited significant differential expression (fold change ≥1.5,* P* ≤0.05) including 368 upregulated lncRNAs and 200 downregulated lncRNAs. The results showed that the expression of XIST, CCAT1, NEAT1, and MALAT1 is top upregulated lncRNAs, and MALAT1 is mostly significantly dysregulated lncRNAs, which is increased by nearly 17-fold in the presence of TGF-*β*2 (Figure 1(a)). To confirm the microarray data, the top four upregulated lncRNAs were detected in human PCO-attached LECs and normal-attached LECs using qRT-PCR. MALAT1 expression was the most increased by nearly 18-fold in human PCO-attached LECs compared with normal attached LECs (Figure 1(b) and Supplementary Figure S1 A, B, C, and D). Consistent with these results, MALAT1 was most significantly upregulated by nearly 14-fold in LECs obtained from patients with anterior polar cataracts compared with patients with nuclear cataracts (Figure 1(c) and Supplementary Figure S1 E, F, G, and H). Next, to further confirm the microarray results and analyze whether TGF-*β*2 upregulates MALAT1 in primary HLECs, we detected the expression of MALAT1 by qRT-PCR. TGF-*β*2 significantly induced MALAT1 overexpression in primary HLECs in a dose-dependent manner and a time-dependent manner (Figures 1(d) and 1(e)).

### 3.2. TGF-*β*2 Induces Overexpression of EMT Markers in Primary HLECs via a MALAT1-Dependent Mechanism

**Figure 2:**
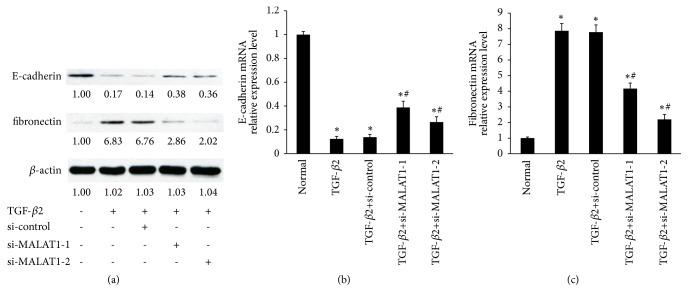
TGF-*β*2 induced the expression of EMT markers in primary HLECs via a MALAT1-dependent mechanism. (a, b, c) The primary HLECs were treated with TGF-*β*2 (5 ng/ml) for 48 h before incubation with MALAT1 siRNAs or control siRNA for 24 h. (a) E-cadherin and fibronectin protein levels in primary HLECs transfected and treated as indicated were detected by Western blot analysis. (b) E-cadherin mRNA levels in primary HLECs were detected by qRT-PCR. (c) Fibronectin mRNA expression in primary HLECs was detected using qRT-PCR. *∗P* < 0.05 compared with normal ((b) and (c)). ^#^*P* < 0.05 compared with group with TGF-*β*2 ((b) and (c)). All of data are presented as the mean± SE of six independent experiments.

The EMT is characterized by downregulation of epithelial differentiation markers (i.e. E-cadherin) and upregulation of mesenchymal cell markers (i.e., fibronectin) [1, 2]. Firstly, consistent with the previous studies, TGF-*β*2 induced EMT of LECs, which significantly inhibited E-cadherin protein and induced fibronectin protein in primary HLECs (Figure 2(a)) [4, 14]. However, TGF-*β*2-induced fibronectin in primary HLECs was suppressed by MALAT1 knockdown (Figure 2(a)). In addition, MALAT1 knockdown ameliorates downregulation of E-cadherin by TGF-*β*2 (Figure 2(a)). Next, TGF-*β*2 inhibited the levels of E-cadherin mRNA in primary HLECs, but the tendency was reversed by MALAT1 siRNA (Figure 2(b)). Moreover, TGF-*β*2 induced the expression of fibronectin mRNA, but these effects were inhibited using MALAT1 siRNA (Figure 2(c)). Taken together, TGF-*β*2 induced the expression of EMT markers in primary HLECs via a MALAT1-dependent mechanism.

### 3.3. Smad4 Is a Target of miR-26a in Primary HLECs

**Figure 3:**
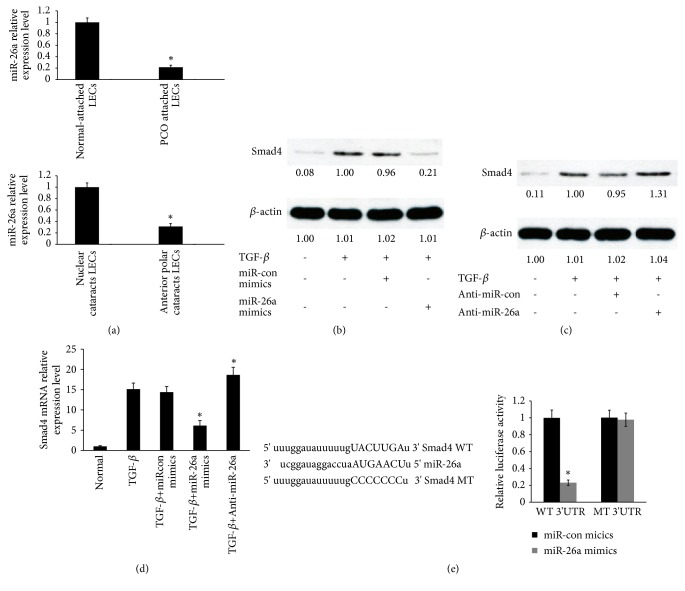
Smad4 is a target of miR-26a in primary HLECs. (a) The levels of miR-26a were detected by qRT-PCR. *∗P* < 0.05 compared with normal-attached LECs or nuclear cataracts. (b) Smad4 protein levels in primary HLECs were detected by Western blot analysis. The primary HLECs were treated with TGF-*β*2 (5 ng/ml) for 48 h before incubation with miR-26a mimics or miR-26a mimics negative control for 6 h. (c) Smad4 protein levels in primary HLECs were detected by Western blot analysis. The primary HLECs were treated with TGF-*β*2 (5 ng/ml) for 48 h before incubation with anti-miR-26a or anti-miR-26a negative control for 6 h. (d) The levels of Smad4 mRNA were detected by qRT-PCR. *∗P* < 0.05 compared with normal or TGF-*β*2 group. (e) The luciferase reporter assays identified that miR-26a directly targets Smad4 in HLECs. *∗P* < 0.05 compared with miR-26a mimics control group. All of data are presented as the mean± SE of six independent experiments.

Our previous studies have identified that miR-26b inhibit EMT of LECs [4]. MiR-26a and miR-26b are members of the miR-26 family [14]. Based on these, we hypothesized miR-26a may be involved in EMT of LECs. Firstly, to confirm these, the levels of miR-26a were determined using qRT-PCR. The current data showed that miR-26a expression was decreased by nearly 5-fold in human PCO-attached LECs compared with normal attached LECs and downregulated by nearly 3-fold in LECs obtained from patients with anterior polar cataracts compared with patients with nuclear cataracts (Figure 3(a) and Supplementary Figure S2 A, B). Next, the protein levels of Smad4 induced by TGF-*β*2 were downregulated in miR-26a-overexpressing primary HLECs (Figure 3(b)). Furthermore, the protein expression of Smad4 induced by TGF-*β*2 was upregulated in primary HLECs treated with anti-miR-26a (Figure 3(c)). In accord with Western blot analysis, qRT-PCR showed that Smad4 mRNA induced by TGF-*β*2 was decreased in miR-26a-overexpressing primary HLECs (Figure 3(d)). Moreover, Smad4 mRNA was increased in primary HLECs treated with anti-miR-26a in the presence of TGF-*β*2 (Figure 3(d)). Importantly, an inverse correlation was found between miR-26a and Smad4 mRNA expression levels in patients (Supplementary Figure S2 C, D). The mechanism that miR-26a downregulated the expression of Smad4 may be miR-26a binding directly to 3'-UTR of Smad4 using miRanda (Figure 3(e)). Finally, we used luciferase reporter assays to demonstrate that miR-26a directly targets Smad4 in HLECs (Figure 3(e)).

### 3.4. MALAT1 Negatively Regulated the Expression of miR-26a

**Figure 4:**
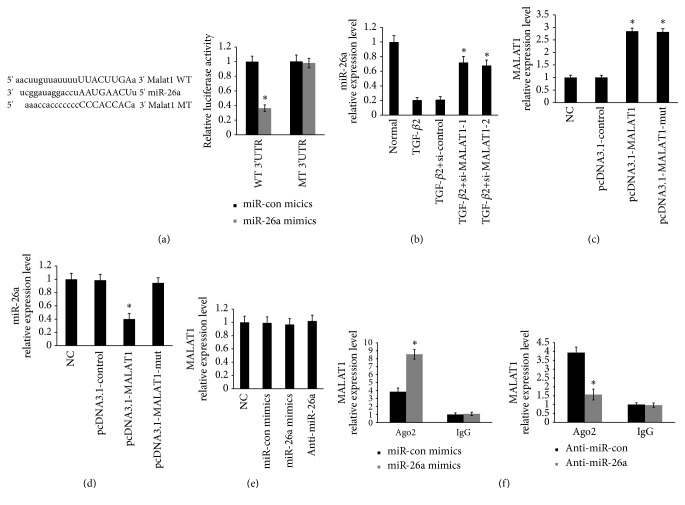
MALAT1 negatively regulated the expression of miR-26a. (a) The luciferase reporter assays identified that MALAT1 contains a binding site for miR-26a in HLECs. *∗P* < 0.05 compared with miR-26a mimics control group. (b) The levels of miR-26a were detected by qRT-PCR. *∗P* < 0.05 compared with TGF-*β*2 group. (c) The levels of MALAT1 were detected by qRT-PCR. *∗P* < 0.05 compared with pcDNA3.1-control group. (d) The levels of miR-26a were detected by qRT-PCR. *∗P* < 0.05 compared with pcDNA3.1-control group or pcDNA3.1-MALAT1-mut group. (e) The levels of MALAT1 were detected by qRT-PCR. (f) The levels of MALAT1 were detected by qRT-PCR. *∗P* < 0.05 compared with miR-26a control group. All of data are presented as the mean± SE of six independent experiments.

LncRNAs and miRNAs usually act as a competing endogenous RNA (ceRNA) [15, 16]. The bioinformatics analysis showed that miR-26a was the potential miRNA which interacts with MALAT1. Next, a dual luciferase reporter assay was performed and confirmed that MALAT1 contains a binding site for miR-26a (Figure 4(a)). Furthermore, the levels of miR-26a were significantly upregulated by knockdown of MALAT1 (Figure 4(b)). To further identify the negative regulation of miR-26a by MALAT1, primary HLECs were treated with pcDNA3.1-MALAT1 vector or pcDNA3.1-MALAT1-mut vector. The expression of MALAT1 was increased by transfecting with the pcDNA3.1-MALAT1 vector and mut vector (Figure 4(c)). The levels of miR-26a were decreased in primary HLECs treated with pcDNA3.1-MALAT1 vector (Figure 4(d)). However, there was no difference from control in the expression of miR-26a when the primary HLECs were treated with the pcDNA3.1-MALAT1-mut vector (mutations in the miRNA-26a response elements) (Figure 4(d)). Moreover, the levels of MALAT1 were unchanged after ectopic expression or knockdown of miR-26a in primary HLECs by treatment with miR-26a mimics and anti-miR-26a (Figure 4(e)). Importantly, an inverse correlation was found between miR-26a and MALAT1 expression levels in patients (Supplementary Figure S3 A, B).

Previous studies have already demonstrated that Ago2 is the only member in AGO family with catalytic activity during the silencing processes of RNA-induced silencing complex (RISC) [22]. Since miRNAs have been demonstrated involved in RNA silencing in an Ago2-dependent manner, we next investigated whether MALAT1 regulated miR-26a in such a manner. To this end, we performed an anti-Ago2 RIP assay on LECs. The endogenous MALAT1 pull-down was specifically enriched in LECs which were transiently overexpressed miR-26a (Figure 4(f)). Furthermore, the endogenous MALAT1 pull-down was decreased in LECs which were transiently anti-miR-26a. Based on these results, we identified that MALAT1 negatively regulated the expression of miR-26a through “sponging” miR-26a.

### 3.5. Knockdown of MALAT1 Inhibits Smad4: A Target of miR-26a

**Figure 5:**
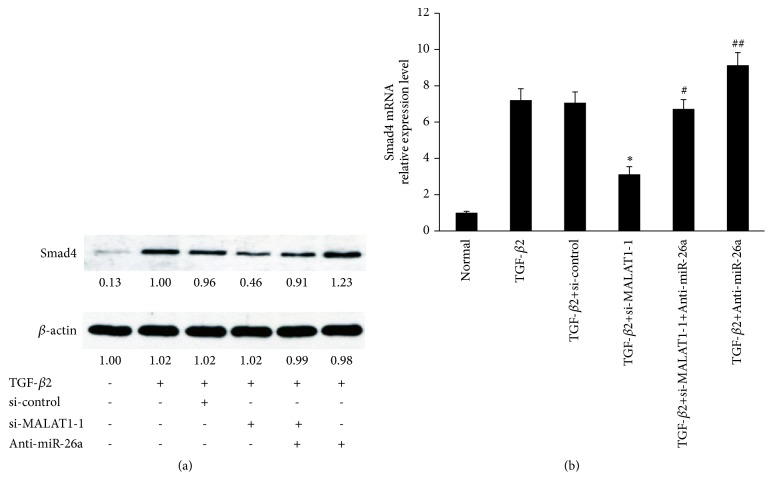
Knockdown of MALAT1 inhibits Smad4, a target of miR-26a. (a) Smad4 protein levels in primary HLECs were detected by Western blot analysis. The primary HLECs were treated with TGF-*β*2 (5 ng/ml) for 48 h before incubation with MALAT1 siRNAs for 24 h or anti-miR-26a for 6 h. (b) The expression levels of Smad4 mRNA were detected by qRT-PCR. The primary HLECs were treated with TGF-*β*2 (5 ng/ml) for 48 h before incubation with MALAT1 siRNAs for 24 h or anti-miR-26a for 6 h. *∗P* < 0.05 compared with normal or TGF-*β*2 group. ^#^*P* < 0.05 compared with TGF-*β*2+MALAT1 siRNAs group. ^##^*P* < 0.05 compared with TGF-*β*2 group and TGF-*β*2+MALAT1 siRNAs group. All of data are presented as the mean± SE of six independent experiments.

Since we have confirmed that MALAT1 negatively regulates miR-26a and miR-26a directly targets Smad4, we next explored whether MALAT1 was involved in Smad4 expression induced by TGF-*β*2 via directly controlling miR-26a in primary HLECs. The results showed that knockdown of MALAT1 significantly downregulated the protein levels of Smad4 induced by TGF-*β*2 (Figure 5(a)). However, anti-miR-26a ameliorated these effects (Figure 5(a)). Next, qRT-PCR also showed similar effects (Figure 5(b)). Taken together, these results indicated that MALAT1 partially controls Smad4 expression induced by TGF-*β*2 via competing with miRNA-26a.

### 3.6. LncRNA MALAT1/Smad4 Axis Is Involved in TGF-*β*2 Induced EMT of LECs

**Figure 6:**
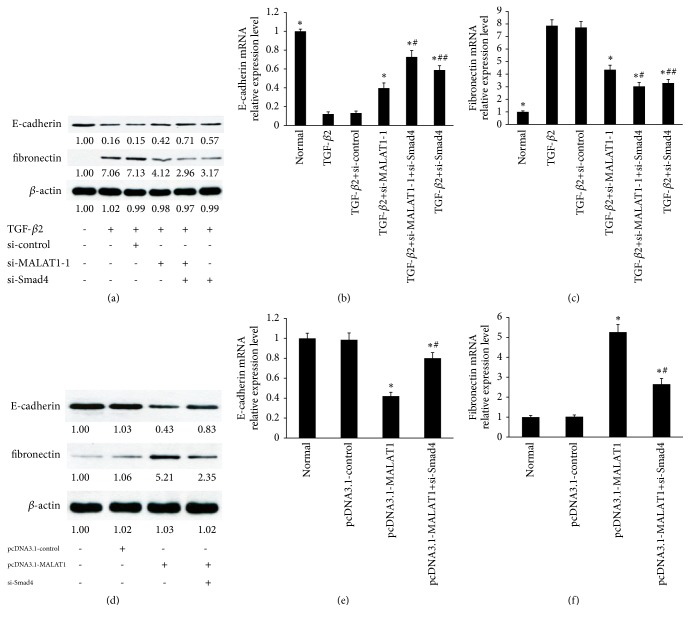
LncRNA MALAT1/Smad4 axis is involved in TGF-*β*2 induced EMT of LECs. (a, b, c) The primary HLECs were treated with TGF-*β*2 (5 ng/ml) for 48 h before incubation with MALAT1 siRNAs or Smad4 siRNA for 24 h. (a) E-cadherin and fibronectin protein levels in primary HLECs were detected by Western blot analysis. (b) E-cadherin mRNA levels in primary HLECs were detected by qRT-PCR. (c) Fibronectin mRNA expression in primary HLECs was detected using qRT-PCR. *∗P* < 0.05 compared with TGF-*β*2 group ((b) and (c)). ^#^*P* < 0.05 compared with TGF-*β*2 group and TGF-*β*2+MALAT1 siRNAs group ((b) and (c)). ^##^*P* < 0.05 compared with TGF-*β*2+MALAT1 siRNAs group and TGF-*β*2+MALAT1 siRNAs+Smad4 siRNAs group ((b) and (c)). (d, e, f) The primary HLECs were treated with TGF-*β*2 (5 ng/ml) for 48 h before incubation with pcDNA3.1-MALAT1 vector or Smad4 siRNA for 24 h. (d) E-cadherin and fibronectin protein levels in primary HLECs were detected by Western blot analysis. (e) E-cadherin mRNA levels in primary HLECs were detected by qRT-PCR. (f) Fibronectin mRNA expression in primary HLECs was detected using qRT-PCR. *∗P* < 0.05 compared with normal group and pcDNA3.1-control group ((e) and (f)). ^#^*P* < 0.05 compared with pcDNA3.1-MALAT1 group ((e) and (f)). All of data are presented as the mean± SE of six independent experiments.

These findings, which indicated that MALAT1 acts as a ceRNA targeting Smad4 by binding miR-26a, prompted us to investigate whether MALAT1/Smad4 axis is involved in TGF-*β*2 induced EMT of LECs. Firstly, TGF-*β*2-induced fibronectin in primary HLECs was suppressed by MALAT1 knockdown and Smad4 knockdown (Figure 6(a)). Next, TGF-*β*2 inhibited the levels of E-cadherin in primary HLECs, but the tendency was reversed by MALAT1 siRNA and Smad4 siRNA (Figure 6(a)). These effects were enhanced by MALAT1 knockdown together with Smad4 knockdown simultaneously. Furthermore, qRT-PCR also showed similar effects (Figures 6(b) and 6(c)).

In addition, the protein expression of fibronectin was increased by transfecting with the pcDNA3.1-MALAT1 vector; these effects were inhibited using Smad4 siRNA (Figure 6(d)). Moreover, the protein expression of E-cadherin was decreased by treatment with the pcDNA3.1-MALAT1 vector, but the tendency was reversed by Smad4 siRNA (Figure 6(d)). Finally, qRT-PCR also showed similar effects (Figures 6(e) and 6(f)). These data indicated that lncRNA MALAT1/Smad4 axis is involved in TGF-*β*2 induced EMT of LECs.

## 4. Discussion

In the present study, our data clearly identified that a total of 568 lncRNAs are differentially expressed in primary HLECs in the presence of TGF-*β*2 and MALAT1 is mostly significantly dysregulated lncRNAs, which is increased by nearly 17-fold. Next, our results showed that TGF-*β*2 induces the expression of EMT markers in primary HLECs via a MALAT1-dependent mechanism. The mechanism is that MALAT1 negatively regulates miR-26a and miR-26a directly targets Smad4. Finally, we demonstrated that MALAT1/miR-26a/Smad4 axis is involved in TGF-*β*2 induced EMT of LECs.

Although lncRNAs are noncoding transcripts and lack protein-coding capability, they play a pivotal role in regulation of gene expression [23, 24]. Especially, accumulating evidence shows that lncRNAs are involved in the process of EMT in the cancer, including bladder cancer, nasopharyngeal carcinoma, breast cancer, gastric cancer, and esophageal cancer [25, 26]. It is well known that EMT of LECs is a crucial cause of PCO formation [1–5]. Importantly, TGF-*β*2 is considered to be an important inducer of EMT-related changes in PCO [19]. In the present study, we firstly confirmed that TGF-*β*2 induces the expression of MALAT1 in the primary HLECs in a dose-dependent manner and a time-dependent manner. Next, we provided further evidence of MALAT1 being significantly upregulated in human PCO-attached LECs and in LECs obtained from patients with anterior polar cataracts. Based on these results, we identified that MALAT1 is involved in the pathogenesis of PCO. Then, to explore whether MALAT1 is involved in TGF-*β*2 induced EMT of LECs, EMT markers, including E-cadherin and fibronectin, were detected. TGF-*β*2-induced fibronectin protein and mRNA were suppressed by MALAT1 knockdown and MALAT1 knockdown ameliorate downregulation of E-cadherin due to TGF-*β*2. Taken together, TGF-*β*2 induced the expression of EMT markers in primary HLECs via a MALAT1-dependent mechanism.

Our previous studies have confirmed that miR-26b inhibit EMT of LECs [4]. Based on the fact that miR-26a and miR-26b are members of the miR-26 family, we hypothesized that miR-26a may be involved in EMT of LECs. Firstly, these results, where miR-26a expression was decreased in human PCO-attached LECs and in LECs obtained from patients with anterior polar cataracts, confirmed that miR-26a is involved in the pathogenesis of PCO. Next, consistent with the previous study, we identified that miR-26a play a crucial role in TGF*β*2-stimulated EMT [14]. During EMT, several transcription factors, such as Smad and Snail, are activated by TGF-*β*2 [27]. The canonical TGF-*β*2/SMAD4 signaling pathway is that TGF-*β*2 activates its receptor and in turn phosphorylates its downstream target SMAD2/3, which hetero-oligomerizes with Smad4 and translocates to the nucleus [27–29]. Then, they recruit cotranscriptional factors to transactivate or repress target genes, which promote transdifferentiation of LECs to attain a mesenchymal phenotype [27–29]. The previous study has confirmed that miR-26a downregulates Smad1 and Smad4 in differentiating myoblasts by directly targeting their 3'UTRs [30]. Consistent with the previous study, we confirmed that miR-26a affect the levels of Smad4 induced by TGF-*β*2 via overexpressing or knockdown of miR-26a in primary HLECs, respectively [30]. The mechanism, which miR-26a downregulated the expression of Smad4, is miR-26a binding directly to 3'-UTR of Smad4 in primary HLECs.

Recently, a study demonstrated that MALAT1 could sponge miR-211 as a competing endogenous RNA to suppress tumor growth and progression in ovarian carcinoma [31]. MALAT1 exerted its oncogenic function in osteosarcoma as a ceRNA to suppress miR-34a expression and upregulate CCND1 [32]. Furthermore, MALAT1 can act as a competing endogenous RNA to modulate miR-124/STAT3 in NSCLC [33]. Enlightened by the ceRNAs regulatory network and based on the fact that MALAT1 contains a target site of miR-26a (Figure 4(a)), we hypothesized that MALAT1 may be as a ceRNA to compete and sink miR-26a expression. It is interesting that Wang and Sun have demonstrated that MALAT1 contains a target site of miR-26a-5p and MALAT1 directly regulates miR-26a-5p in osteosarcoma cells [34]. Consistent with the previous studies, the current data also confirmed that MALAT1 negatively regulated the expression of miR-26a through “sponging” miR-26a in primary HLECs. Finally, we identified that MALAT1/miR-26a/Smad4 axis is involved in TGF-*β*2 induced EMT of LECs.

## 5. Conclusion

In summary, the current study showed that TGF-*β*2 induces EMT in primary HLECs via a MALAT1-dependent mechanism. The mechanism is that MALAT1 negatively regulates the expression of miR-26a through “sponging” miR-26a and in turn induces Smad4. Therefore, these results also indicated that MALAT1/miR-26a/Smad4 axis is involved in TGF-*β*2 induced EMT of LECs and MALAT1 is a potential therapeutic target for the treatment of PCO.

## Figures and Tables

**Table 1:** The sequences used for MALAT1 siRNA.

	*Sequence*	
	sense	antisense
siMALAT1-1	5'-CACAGGGAAAGCGAGTGGTTGGTAA-3'	5'-TTACCAACCACTCGCTTTCCCTGTG-3'
siMALAT1-2	5'-GAGGUGUAAAGGGAUUUAUTT-3'	5'-AUAAAUCCCUUUACACCUCTT-3'
si-control	5'-UUCUCCGAACGUGUCACGUTT-3'	5'-ACGUGACACGUUCGGAGAATT-3'

**Table 2:** Primers used for qRT-PCR.

*Primers*	*Sequence*	
	sense	antisense
GAPDH	5'-AGGTCGGTGTGAACGGATTTG-3'	5'-TGTAGACCATGTAGTTGAGGTCA-3'
U6	5'-CTCGCTTCGGCAGCACA-3'	5'-AACGCTTCACGAATTTGCGT-3'
miR-26a	5'-TTGGATCCGTCAGAAATTCTCTCCCGAGG -3'	5'-GGTCTAGATGTGAACTCTGGTGTTGGTGC -3'
MALAT1	5'-AAAGCAAGGTCTCCCCACAAG-3'	5'-GGTCTGTGCTAGATCAAAAGGCA-3'
XIST	5'-AAGGTCTTGCCGCAGTGTAA-3'	5'-ATGGAGGGAGGTTCAGACCA-3'
CCAT1	5'-TTTATGCTTGAGCCTTGA-3'	5'-CTTGCCTGAAATACTTGC-3'
NEAT1	5'-TTTGTGCTTGGAACCTTGCT-3'	5'-TCAACGCCCCAAGTTATTTC-3'
E-cadherin	5'-CGAGAGCTACACGTTCACGG-3'	5'-GGGTGTCGAGGGAAAAATAGG-3'
fibronectin	5'-TCTGTGCCTCCTATCTATGTGC-3'	5'-GAGGGACCACGACAACTCTTC-3'
Smad4	5'-CGGACATTACTGGCCTGTTC-3'	5'-TAGGGCAGCTTGAAGGAAACC-3'

## Data Availability

All relevant data used to support the findings of this study are included within the article.

## Supplementary Materials

Supplementary Materials Supplementary Figure S1: lncRNAs were overexpression in human PCO-attached LECs and LECs obtained from patients with anterior polar cataracts. (A, B, C, and D). The expression of XIST, CCAT1, NEAT1, and MALAT1 was significantly increased in human PCO-attached LECs (n=16) compared with normal attached LECs (n=15). *∗*P < 0.05 compared with PCO attached LECs. (E, F, G, H) The expression of XIST, CCAT1, NEAT1, and MALAT1 was significantly increased in LECs obtained from patients with anterior polar cataracts (n=33) compared with patients with nuclear cataracts (n=33). *∗*P < 0.05 compared with anterior polar cataracts. All of data are presented as the mean± SE of six independent experiments. Supplementary Figure S2: Smad4 is a target of miR-26a in primary HLECs. (A) miR-26a expression was significantly decreased in human PCO-attached LECs (n=16) compared with normal attached LECs (n=15). *∗*P < 0.05 compared with PCO attached LECs. (B) The expression of miR-26a was significantly decreased in LECs obtained from patients with anterior polar cataracts (n=33) compared with patients with nuclear cataracts (n=33). *∗*P < 0.05 compared with anterior polar cataracts. (C) The correlation between miR-26a and Smad4 mRNA expression was detected in human PCO-attached LECs and normal-attached LEC samples by Pearson correlation analysis. n=31. (D) The correlation between miR-26a and Smad4 mRNA expression was detected in LECs obtained from patients with anterior polar cataracts and patients with nuclear cataracts by Pearson correlation analysis. n=66. All of data are presented as the mean± SE of six independent experiments. Supplementary Figure S3: MALAT1 and miR-26a expression in patients was an inverse correlation. (A) The correlation between MALAT1 and miR-26a expression was detected in human PCO-attached LECs and normal-attached LEC samples by Pearson correlation analysis. n=31. (D) The correlation between MALAT1 and miR-26a expression was detected in LECs obtained from patients with anterior polar cataracts and patients with nuclear cataracts by Pearson correlation analysis. n=66.
