# Supplementary material for: Long Noncoding RNA MALAT1 Acts as a Competing Endogenous RNA to Regulate TGF-β2 Induced Epithelial-Mesenchymal Transition of Lens Epithelial Cells by a MicroRNA-26a-Dependent Mechanism
Source: Biomed Res Int. 2019 Apr 22;2019:1569638. doi: 10.1155/2019/1569638 (PMC6501259; doi:10.1155/2019/1569638)
Supplement: Supplementary Materials — Supplementary Figure S1: lncRNAs were overexpression in human PCO-attached LECs and LECs obtained from patients with anterior polar cataracts. (A, B, C, and D). The expression of XIST, CCAT1, NEAT1, and MALAT1 was significantly increased in human PCO-attached LECs (n=16) compared with normal attached LECs (n=15). ∗P < 0.05 compared with PCO attached LECs. (E, F, G, H) The expression of XIST, CCAT1, NEAT1, and MALAT1 was significantly increased in LECs obtained from patients with anterior polar cataracts (n=33) compared with patients with nuclear cataracts (n=33). ∗P < 0.05 compared with anterior polar cataracts. All of data are presented as the mean± SE of six independent experiments. Supplementary Figure S2: Smad4 is a target of miR-26a in primary HLECs. (A) miR-26a expression was significantly decreased in human PCO-attached LECs (n=16) compared with normal attached LECs (n=15). ∗P < 0.05 compared with PCO attached LECs. (B) The expression of miR-26a was significantly decreased in LECs obtained from patients with anterior polar cataracts (n=33) compared with patients with nuclear cataracts (n=33). ∗P < 0.05 compared with anterior polar cataracts. (C) The correlation between miR-26a and Smad4 mRNA expression was detected in human PCO-attached LECs and normal-attached LEC samples by Pearson correlation analysis. n=31. (D) The correlation between miR-26a and Smad4 mRNA expression was detected in LECs obtained from patients with anterior polar cataracts and patients with nuclear cataracts by Pearson correlation analysis. n=66. All of data are presented as the mean± SE of six independent experiments. Supplementary Figure S3: MALAT1 and miR-26a expression in patients was an inverse correlation. (A) The correlation between MALAT1 and miR-26a expression was detected in human PCO-attached LECs and normal-attached LEC samples by Pearson correlation analysis. n=31. (D) The correlation between MALAT1 and miR-26a expression was detected in LECs obtained from patients with ante [file 1569638.f1.doc]

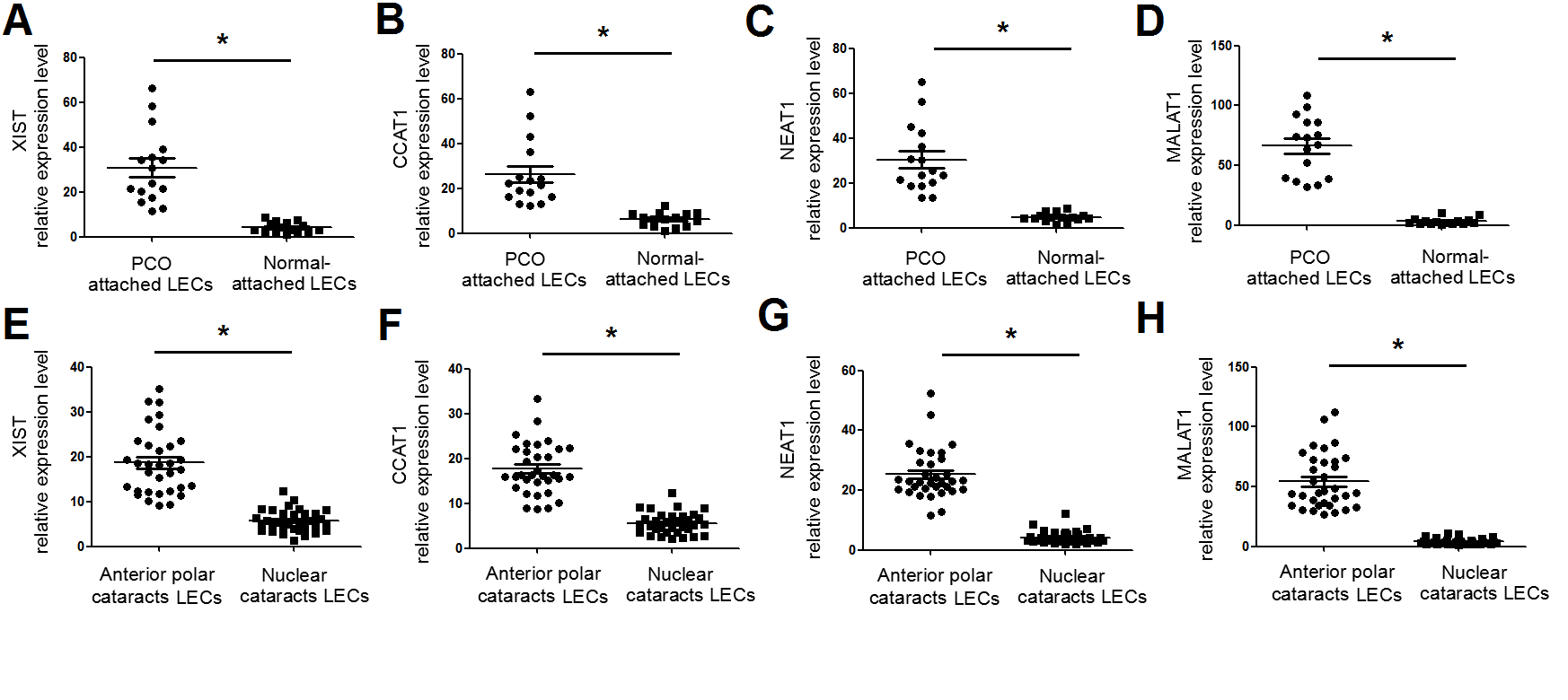


Supplementary Figure S1: lncRNAs were over-expression in human PCO-attached LECs and LECs obtained from patients with anterior polar cataracts. (A, B, C, D) The expression of XIST, CCAT1, NEAT1, and MALAT1 was significantly increased in human PCO-attached LECs (n=16) compared with normal attached LECs (n=15). **P* < 0.05 compared with PCO attached LECs. (E, F, G, H) The expression of XIST, CCAT1, NEAT1, and MALAT1 was significantly increased in LECs obtained from patients with anterior polar cataracts (n=33) compared with patients with nuclear cataracts (n=33). **P* < 0.05 compared with anterior polar cataracts. All of data are presented as the mean± SE of six independent experiments.


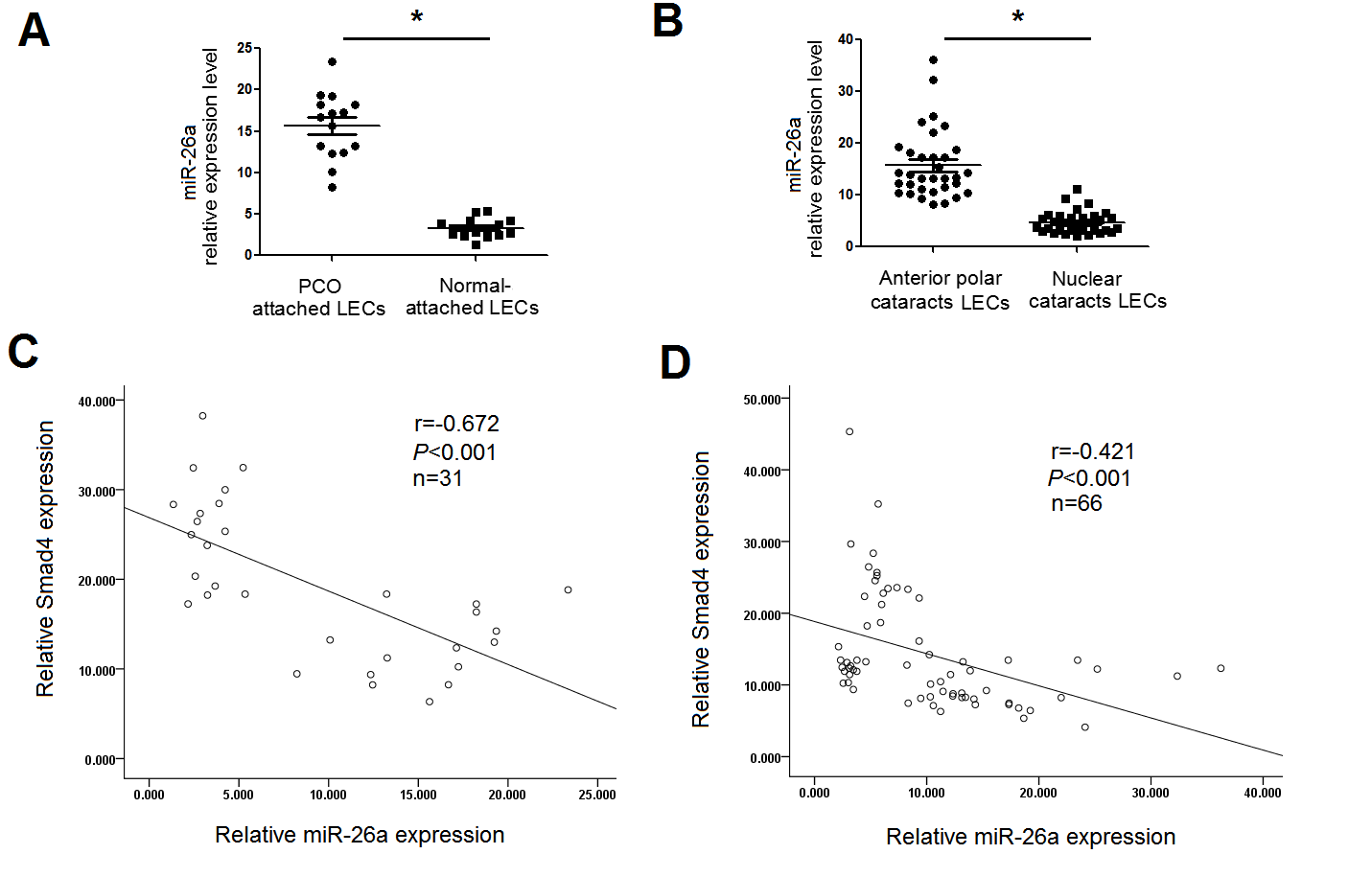


Supplementary Figure S2: Smad4 is a target of miR-26a in primary HLECs. (A) miR-26a expression was significantly decreased in human PCO-attached LECs (n=16) compared with normal attached LECs (n=15). **P* < 0.05 compared with PCO attached LECs. (B) The expression of miR-26a was significantly decreased in LECs obtained from patients with anterior polar cataracts (n=33) compared with patients with nuclear cataracts (n=33). **P* < 0.05 compared with anterior polar cataracts. (C) The correlation between miR-26a and Smad4 mRNA expression was detected in human PCO-attached LECs and normal-attached LEC samples by Pearson correlation analysis. n=31. (D) The correlation between miR-26a and Smad4 mRNA expression was detected in LECs obtained from patients with anterior polar cataracts and patients with nuclear cataracts by Pearson correlation analysis. n=66. All of data are presented as the mean± SE of six independent experiments.


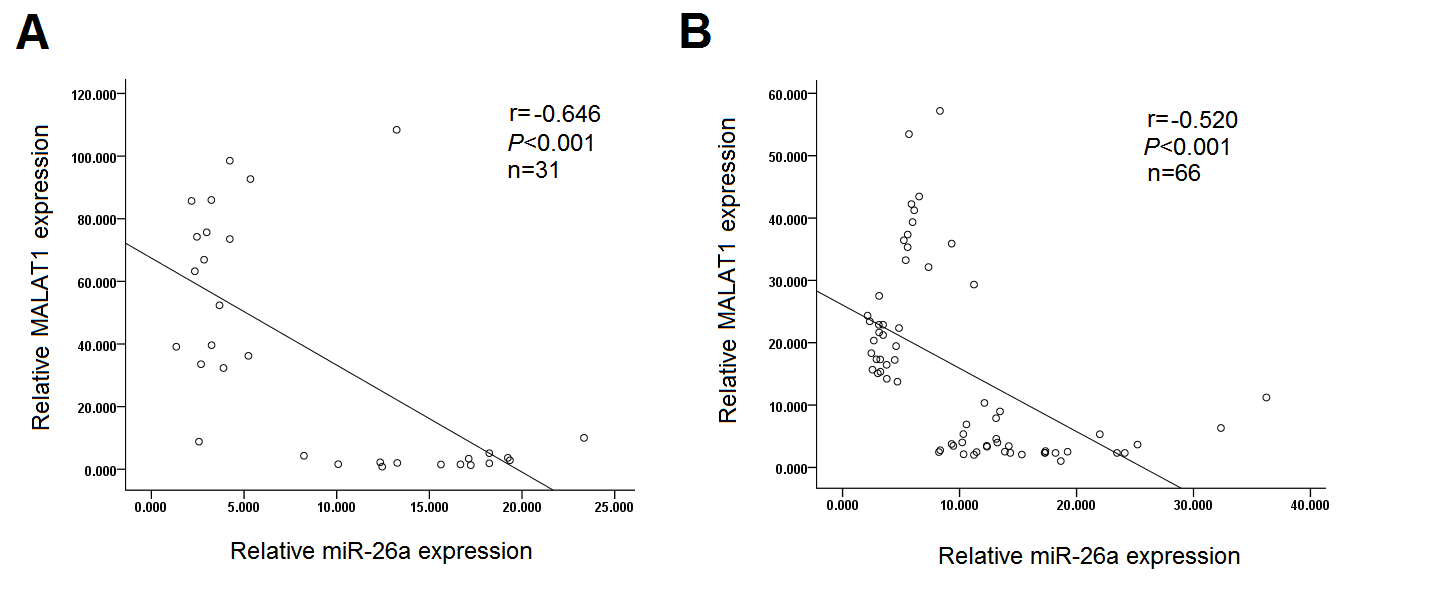


Supplementary Figure S3: MALAT1 and miR-26a expression in patients was an inverse correlation. (A) The correlation between MALAT1 and miR-26a expression was detected in human PCO-attached LECs and normal-attached LEC samples by Pearson correlation analysis. n=31. (D) The correlation between MALAT1 and miR-26a expression was detected in LECs obtained from patients with anterior polar cataracts and patients with nuclear cataracts by Pearson correlation analysis. n=66.
